# Regional Citrate Anticoagulation in Continuous Renal Replacement Therapy: Is Metabolic Fear the Enemy of Logic? A Systematic Review and Meta-Analysis of Randomised Controlled Trials

**DOI:** 10.3390/life13051198

**Published:** 2023-05-17

**Authors:** Rita Jacobs, Walter Verbrugghe, Karolien Dams, Ella Roelant, Marie Madeleine Couttenye, Dirk Devroey, Philippe Jorens

**Affiliations:** 1 Intensive Care Department, Antwerp University Hospital, 2650 Edegem, Belgium; 2 Clinical Trial Center (CTC), Antwerp University Hospital, 2650 Edegem, Belgium; 3 Department of Nephrology and Hypertension, Antwerp University Hospital, 2650 Edegem, Belgium; 4 Laboratory of Experimental Medicine and Pediatrics (LEMP), 2000 Antwerpen, Belgium; 5 Deparmtment of Family Medicine and Chronic Care, Faculty of Medicine and Framacy, Vrije Universiteit Brussels (VUB), 1090 Brussels, Belgium

**Keywords:** citrate, heparin, anticoagulation, continuous renal replacement therapy, metabolic complications, filter lifespan

## Abstract

Background: Anticoagulation is recommended to maintain the patency of the circuit in continuous renal replacement therapy (CRRT). However, anticoagulation-associated complications can occur. We performed a systematic review and meta-analysis to compare the efficacy and safety of citrate anticoagulation to heparin anticoagulation in critically ill patients treated with CRRT. Methods: Randomised controlled trials (RCTs) evaluating the safety and efficacy of citrate anticoagulation and heparin in CRRT were included. Articles not describing the incidence of metabolic and/or electrolyte disturbances induced by the anticoagulation strategy were excluded. The PubMed, Embase, and MEDLINE electronic databases were searched. The last search was performed on 18 February 2022. Results: Twelve articles comprising 1592 patients met the inclusion criteria. There was no significant difference between the groups in the development of metabolic alkalosis (RR = 1.46; (95% CI (0.52–4.11); *p* = 0.470)) or metabolic acidosis (RR = 1.71, (95% CI (0.99–2.93); *p* = 0.054)). Patients in the citrate group developed hypocalcaemia more frequently (RR = 3.81; 95% CI (1.67–8.66); *p* = 0.001). Bleeding complications in patients randomised to the citrate group were significantly lower than those in the heparin group (RR 0.32 (95% CI (0.22–0.47); *p* < 0.0001)). Citrate showed a significantly longer filter lifespan of 14.52 h (95% CI (7.22–21.83); *p* < 0.0001), compared to heparin. There was no significant difference between the groups for 28-day mortality (RR = 1.08 (95% CI (0.89–1.31); *p* = 0.424) or 90-day mortality (RR 0.9 (95% CI (0.8–1.02); *p* = 0.110). Conclusion: regional citrate anticoagulation is a safe anticoagulant for critically ill patients who require CRRT, as no significant differences were found in metabolic complications between the groups. Additionally, citrate has a lower risk of bleeding and circuit loss than heparin.

## 1. Introduction

Continuous renal replacement therapy (CRRT) is the treatment of choice for acute kidney injury (AKI) in critically ill patients due to its haemodynamic stability [1]. To prevent clotting of the extracorporeal circuit, anticoagulation is needed. The Kidney Disease Improving Global Outcomes (KDIGO) guidelines recommend regional citrate anticoagulation (RCA) rather than heparin in patients who do not have contraindications for the use of citrate, due to the risk of bleeding and heparin-induced thrombocytopenia (HIT) [2]. Citrate induces anticoagulation by chelating ionized calcium (iCa^++^), an essential component of the coagulation cascade [3]. In RCA-CRRT, different forms of citrate solutions are available. They can be classified as high- or low-citrate concentration solutions [4] and are characterized by hypertonicity and isotonicity in sodium [5]. The advantage of RCA is that apart from being an anticoagulant with a reduced risk of bleeding, it also acts as a buffer [3].

However, due to the high sodium and citrate content of the citrate solutions used and the loss of calcium bound to citrate in the effluent, metabolic abnormalities can arise [6]. The acid–base disturbances associated with citrate anticoagulation include metabolic alkalosis due to the metabolism of an increasing citrate load returning to the patient. On the other hand, if citrate is insufficiently metabolized, it acts as a weak acid, and patients can become acidotic [7,8,9]. The increased risk of citrate accumulation in patients is the main drawback of RCA-CRRT. Electrolyte abnormalities of CRRT can be divided into two broad categories: (1) those caused by the removal of electrolytes by dialysis or haemofiltration with inadequate replacement (hypophosphatemia, hypokalaemia, hypocalcaemia, hypomagnesemia), and (2) those caused using trisodium citrate as the anticoagulant (hypernatremia, hypercalcaemia, hypocalcaemia) [10].

A recent Cochrane review of pharmacological interventions for preventing clotting of extracorporeal circuits during CRRT could not show superiority of one anticoagulant over another [11]. Thus, it remains unclear whether citrate results in better filter survival, but with an increased risk of metabolic complications. The aim of our systematic review and meta-analysis was to summarize the available data on the efficacy and safety of RCA anticoagulation for CRRT.

## 2. Materials and Methods

### 2.1. Search Strategy

The protocol for the present systematic review and meta-analysis has been registered at PROSPERO (ID = CRD42022330031). In accordance with the Preferred Reporting Items for Systematic Reviews and Meta-analyses (PRISMA) statement [12], we performed a comprehensive search of the PubMed, Embase, and Cochrane Library databases. We searched by using the following MeSH terms: regional citrate anticoagulation and continuous renal replacement therapy and adverse effects. Furthermore, we manually searched the reference lists of the retrieved studies and reviewed articles for additional publications. The search was last performed on 18 February 2022, and the search was restricted to the English language.

### 2.2. Study Selection

Two reviewers performed the search independently, and disagreements were discussed with a third reviewer. The inclusion criteria were as follows: (i) adult (age > 18 years) patients, (ii) randomised controlled trials (RCTs) comparing RCA with unfractionated heparin (UH) or low-molecular-weight heparin (LMWH), (iii) the modality of renal replacement therapy (RRT) was continuous, (iv) safety-related outcomes, such as metabolic and/or electrolyte disturbances, were reported, and (v) efficacy outcomes by the means of filter lifespan (FLS) were investigated. The exclusion criteria were as follows: (i) full text was not available, (ii) studies that did not analyse the safety and efficacy of RCA, (iii) no data on metabolic effect were available, and (iv) review articles, case reports, letters, editorials, conference abstracts, and comments.

### 2.3. Study Quality

The risk of bias of the included articles was assessed through the Cochrane Handbook for Systematic Reviews of Interventions version 6.3, regarding bias arising from the randomisation process, whether the allocation sequence was random and adequately concealed, and if there were baseline differences between the intervention groups. We examined whether there were deviations from intended interventions, bias due to missing outcome data, bias in measurement of the outcome, and bias in selection of the reported result [13].

### 2.4. Grading Quality of Evidence

The quality of evidence for outcomes was assessed according to the Grading of Recommendations Assessment, Development and Evaluation (GRADE) Working Group criteria [14]. This is based on the risk of bias, inconsistency of results, indirectness of evidence, imprecision, and publication bias. The quality of evidence was categorized as high, moderate, low, or very low.

### 2.5. Data Collection

Two authors (RJ and WV) independently extracted data from the included articles using customized data abstraction forms in the Systematic Review Data Repository (SDRD) online platform. The following data from the included papers were recorded: (i) the first author, published year, and study type, (ii) sample size, subject ages, and follow-up period, (iii) inclusion and exclusion criteria, (iv) endpoints, (v) composition of citrate and replacement fluids, (vi) other coagulation method, (vii) characteristics of the CRRT prescription, (viii) mean filter lifespan, (ix) metabolic complications, (x) electrolyte disturbances, (xi) citrate accumulation, (xii) other adverse events, i.e., bleeding, HIT, transfusion, (xiii) renal recovery and CRRT days, and (xiv) mortality.

### 2.6. Statistical Analysis

R version 4.1.2 was used for the meta-analysis of the combined statistical data, and a forest map was drawn. To compare the groups for the binary variables, a relative risk was calculated, and for the continuous variables, a difference in medians was used. For the binary variables, the inverse variance method was used with logit transformation of proportions, and the DerSimonian–Laird estimator for between-study variance tau-squared. In the case of zero events, the hybrid correction of Wei et al. (2021) was applied [15]. For the continuous outcomes, the quantile estimation (QE) of McGrath et al. (2020) was used [16]. The heterogeneity of the included studies was evaluated with different measures, such as the Q-test, the H and the I2 measure. In cases of homogeneity (H = 1, I2 < 50%), the fixed effects model was used for the meta-analysis; otherwise, the random-effects model was used to pool the summary measures. Trial sequential analysis was performed to calculate monitoring boundaries to evaluate the accumulating evidence taking into account the repeated significance testing. For this, TSA version 0.9.5.10 beta (http://www.ctu.dk/tsa accessed on 1 February 2023) was used.

## 3. Results

### 3.1. Selection of Studies

**Figure 1:**
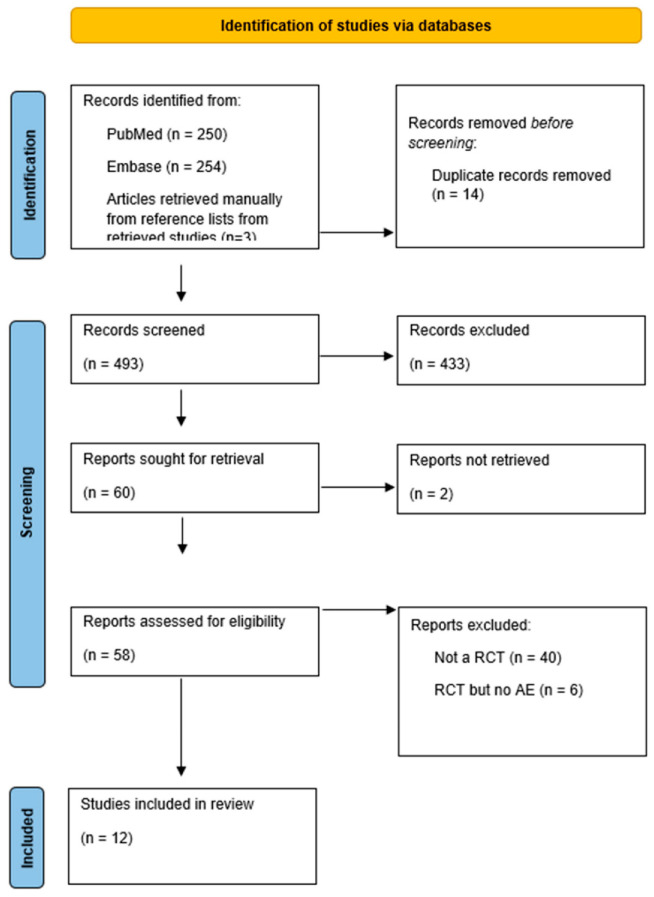
Prisma flowchart.

The study inclusion flow chart is shown in Figure 1. The search initially yielded 507 references. Of these, 14 duplicate publications were excluded. After screening, based on the titles and abstracts, 60 potentially relevant papers remained. A total of 48 articles were then excluded for the following reasons: two articles were excluded because they could not be retrieved, six articles did not meet our inclusion criteria since they did not report acid–base abnormalities, and 40 articles were excluded because they were not RCTs. Ultimately, 12 studies with 1592 patients were included in this systematic review (Figure 1, PRISMA flowchart).

### 3.2. Description of the Included Studies and Patients

The main characteristics of the 12 included studies and 1592 patients’ demographic data are summarized in Table 1. All included studies were randomised controlled trials, 10 studies were parallel RCTs [17,18,19,20,21,22,23,24,25,26], and two were crossover RCTs [27,28]. Of these, four were multicentre [18,19,20,21] and eight were single-centre [17,22,23,24,25,26,27,28] studies. The detailed protocols of RCA-CRRT in these studies are shown in Table 2, and Table 3 shows the characteristics of the applied CRRT. The modality of CRRT used was continuous veno–venous haemofiltration (CVVH) in seven studies [19,21,23,24,25,27,28], continuous veno–venous haemodiafiltration (CVVHDF) in three studies [18,22,26], CVVH and continuous veno–venous haemodialysis (CVVHD) in one study [20], and CVVH, CVVHD, and CVVHDF in one study [17]. Replacement fluids in the predilution mode were used in six studies [18,19,20,21,24,27] and in postdilution setup in five studies [22,23,25,26,28]. In one study [17], the dilution mode was different at each study site. Ten studies compared RCA with unfractionated heparin [17,18,19,20,21,22,23,26,27,28], and two studies reported RCA versus low-molecular-weight heparin (LMWH) [24,25]. The studies were published between 2004 and 2020, and had sample sizes ranging from 10 to 596. All trials evaluated patients with AKI who required renal replacement therapy. Patients with liver failure, a high risk of bleeding, severe coagulation disorders, and HIT were excluded in most trials. Baseline characteristics were similar between the treatment groups.

### 3.3. Quality Evaluation

**Figure 2:**
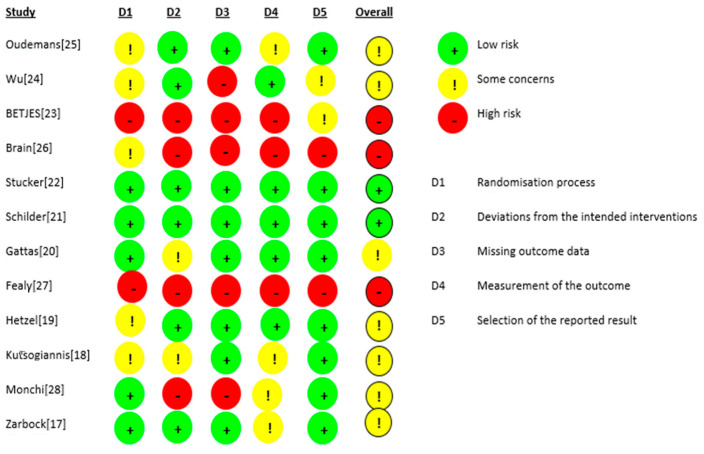

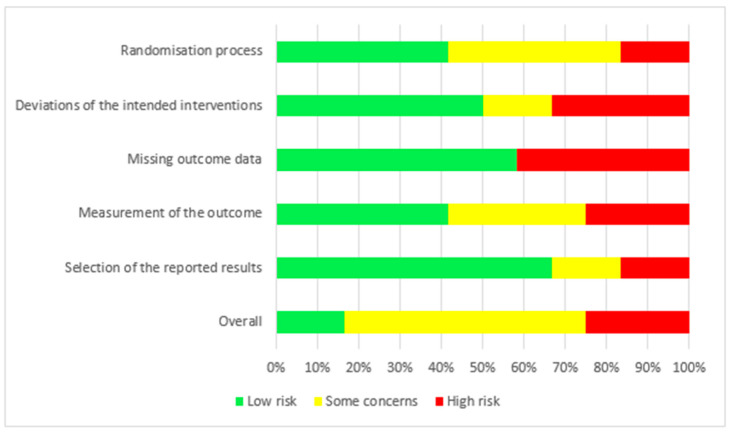
Risk of bias.

The risk of bias for the 12 RCTs is shown in Figure 2. We judged two studies [21,22] as having a low risk of bias. For seven studies, there were some concerns of bias: three studies [18,24,25] had a risk of bias due to allocation concealment, and one study [19] had a risk of bias because the randomisation process was not adequately described. Some outcome data were not available in two studies [17,28]. Three studies were judged to be at a high risk of bias: for the study by Fealy et al. [27], the method of randomisation was not reported in a crossover open-label trial, and there were missing outcome data for two patients. In the study by Betjes et al. [23], the allocation was not concealed, the study was not blinded, and the outcome data showed multiple enrolments and no detailed information. In the RCT by Brain et al. [26], the allocation was not concealed, and the study was not blinded. Baseline characteristics were not the same, i.e., patients in the citrate group were significantly older and sicker (higher APACHE III-j score and more septic shock), and some safety outcome data were not reported. Additionally, due to the nature of the interventions, all 12 studies were not blinded. The GRADE Working Group grades of evidence were low for our safety and efficacy outcomes. This was mainly due to the risk of bias as a result of the lack of blinding and the small population sizes. The full GRADE profiles for the included evidence are shown in Table S1(supplementary material) .

### 3.4. Safety

#### 3.4.1. Acid–Base Status

There was no significant difference between the groups in the development of metabolic alkalosis (RR = 1.46; (95% CI (0.52–4.11); *p* = 0.470)) or metabolic acidosis (RR = 1.7 (95% CI (0.99–2.93); *p* = 0.054)). For the risk of metabolic acidosis, the fixed-effect model of trial sequential analysis was used.

A diversity-adjusted information size of 24,242 participants was calculated based on a metabolic acidosis rate of 2.86% in the heparin group, relative risk reduction of 20%, alpha = 5% (two-sided), beta = 20%, I^2^ = 0%. The cumulative Z-curve crosses the conventional boundary for benefit (in favour of heparin). However, the trial sequential boundary is not crossed, hence more studies are needed.

**Figure 3:**
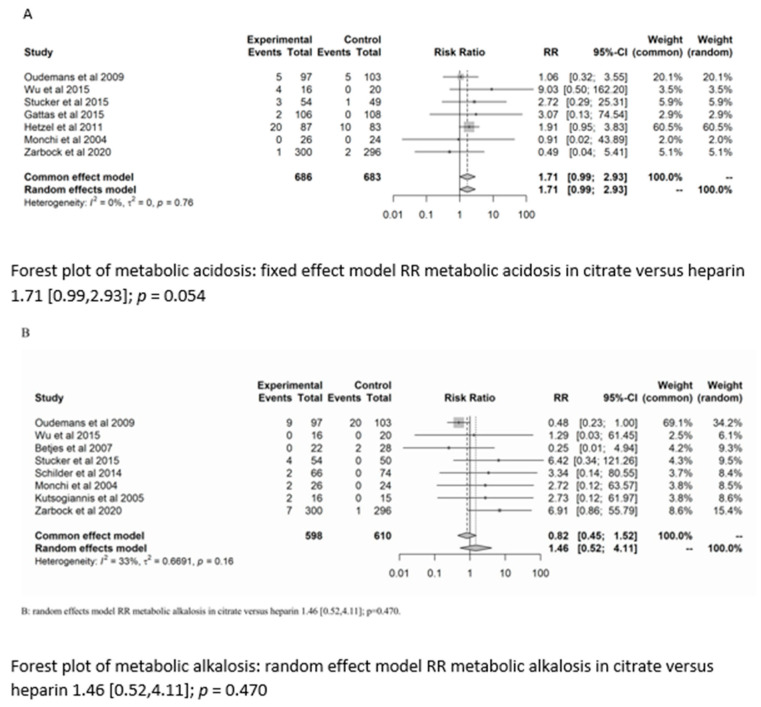

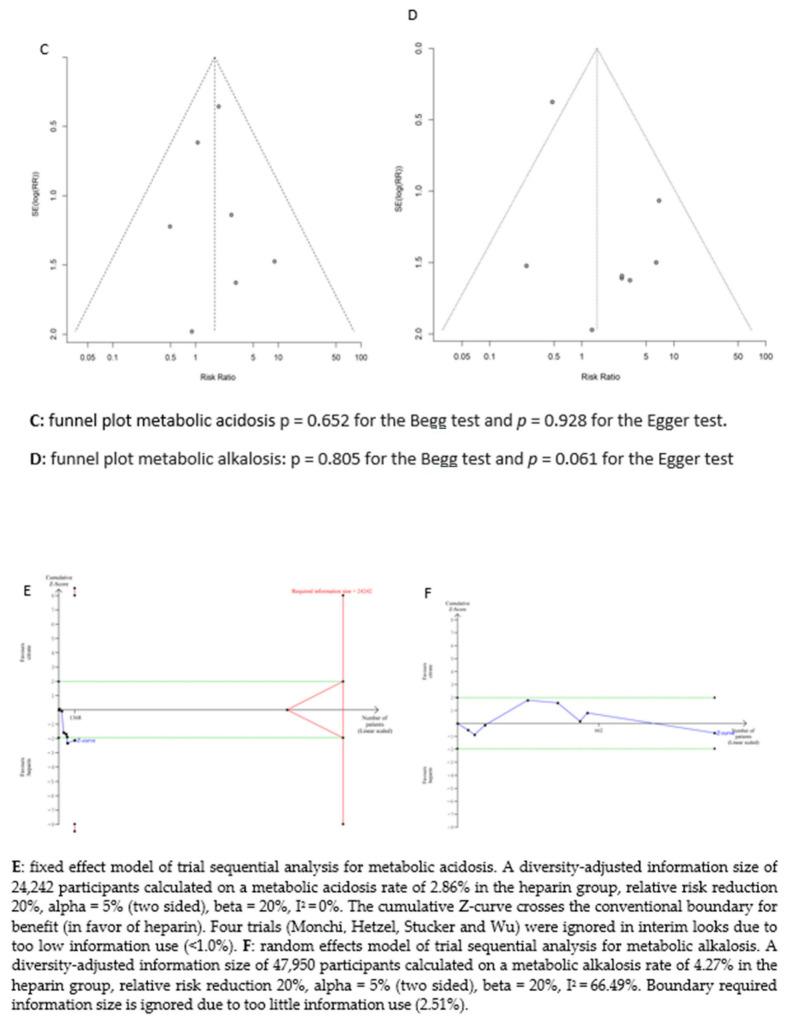
Forest plots, funnel plots and TSA of acid-base disorders.

The random-effects model of trial sequential analysis for metabolic alkalosis was used based on a metabolic alkalosis rate of 4.27% in the heparin group, relative risk reduction of 20%, alpha = 5% (two-sided), beta = 20%, I^2^ = 66.49%, and a diversity-adjusted information size of 47,950 participants was calculated. However, trial sequential analysis could not be performed due to too little data. (see Figure 3).

#### 3.4.2. Electrolyte Disturbances

Patients in the citrate group developed hypocalcaemia more frequently (RR = 3.81; 95% CI (1.67–8.66); *p* = 0.001), while the incidence of hypernatremia (RR = 1.53; 95% CI (0.49–4.78); *p* = 0.467) and hypercalcaemia (RR = 1.8; 95% CI (0.22–14.43); *p* = 0.582) did not differ significantly between the groups. The fixed-effect model of trial sequential analysis was used for hypocalcaemia. A diversity-adjusted information size of 64,219 participants was calculated because of a hypocalcaemia rate of 1.11% in the heparin group, relative risk reduction of 20%, alpha = 5% (two-sided), beta = 20%, I^2^ = 0%. The cumulative Z-curve crosses the conventional boundary for benefit in favour of heparin; however, trial sequential analysis could not be executed due to too insufficient information. This was also the case for hypernatremia and hypercalcaemia. (see Figure S1; supplementary file)

#### 3.4.3. Citrate Accumulation

Six studies [17,19,20,21,22,25] reported the number of patients with citrate accumulation, which was identified by an increased ratio of totCa/iCa^++^. The risk of citrate accumulation was low in both anticoagulation groups, with an RR of 1.83 (95% CI (0.40–8.38); *p* = 0.438).

The random-effects model of trial sequential analysis calculated a diversity-adjusted information size of 82,586 participants based on a citrate accumulation rate of 1.89% in the heparin group, relative risk reduction of 20%, alpha = 5% (two-sided), beta = 20%, I^2^ = 56.01%. Trial sequential analysis was ignored due to too little information. (see Figure S2; supplementary file).

#### 3.4.4. Bleeding

**Figure 4:**
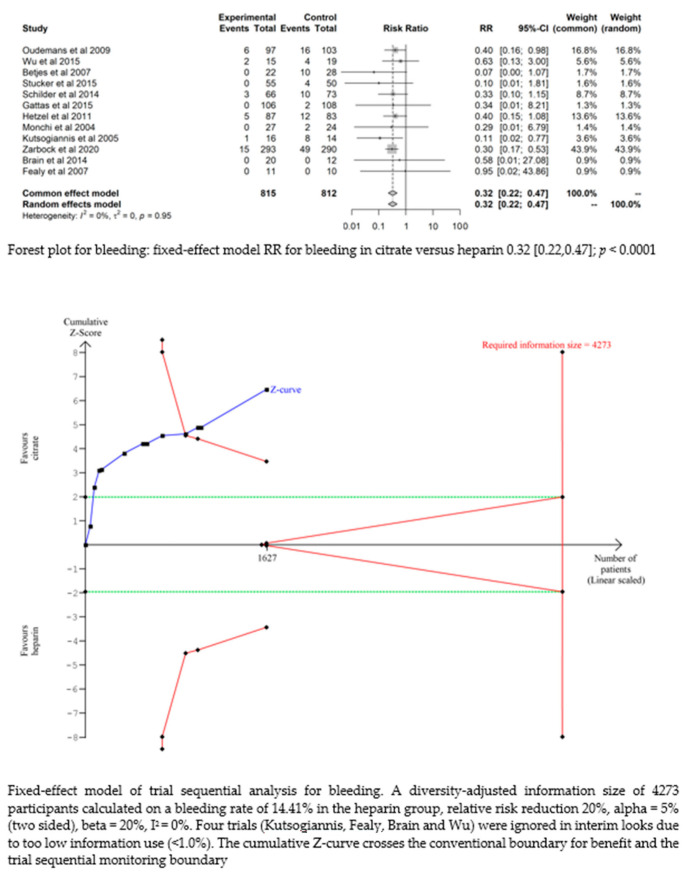

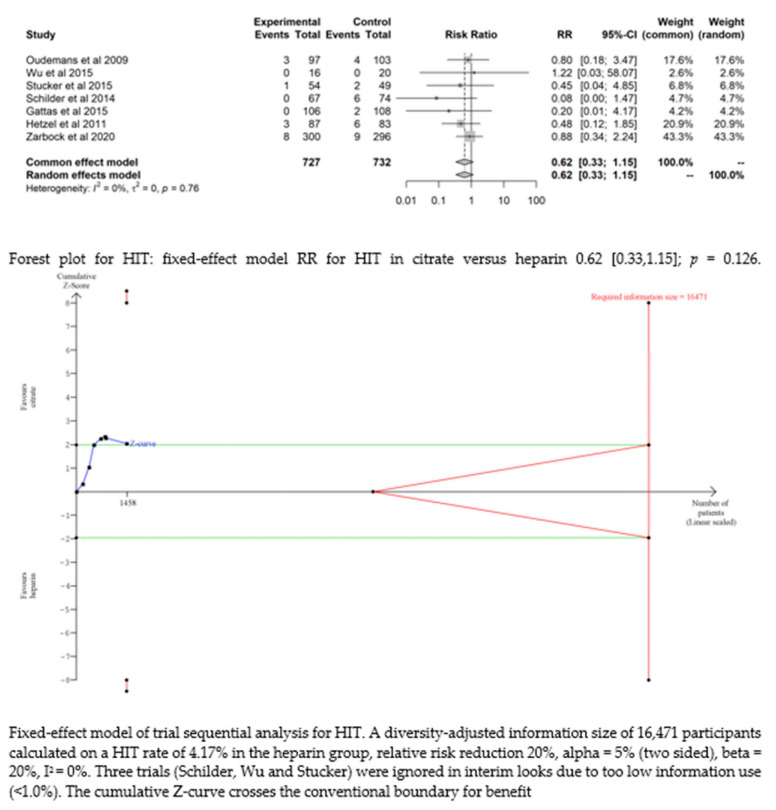

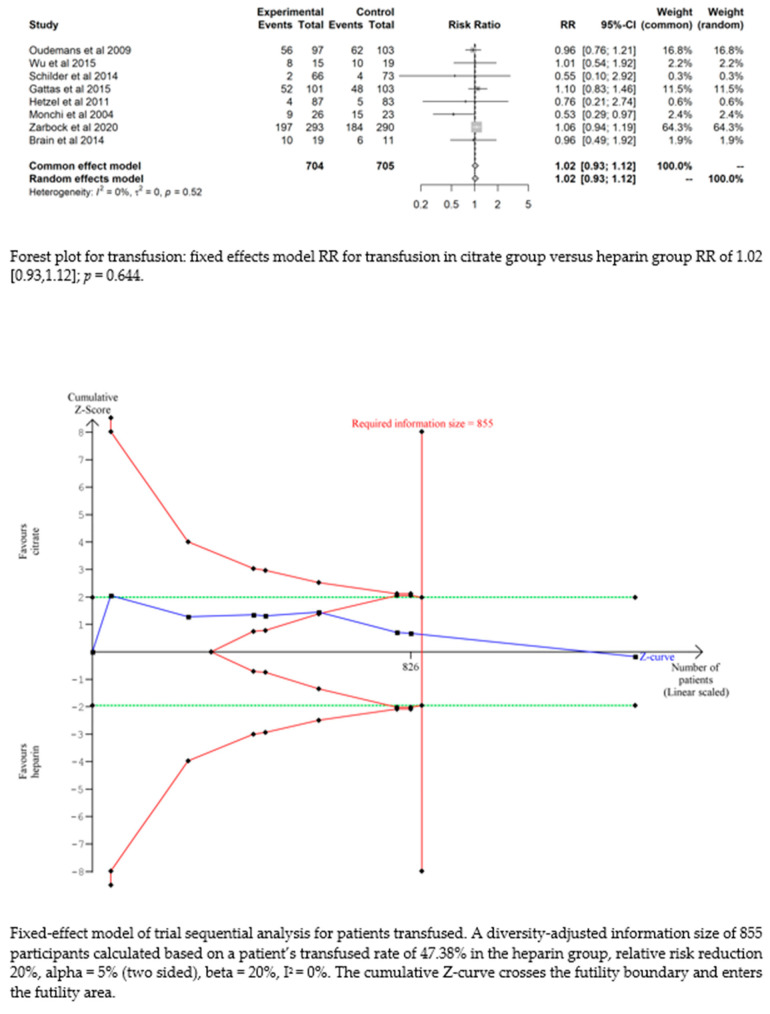
Forest plots and TSA of bleeding, HIT and transfusion.

Bleeding complications in patients randomised to the citrate group were significantly lower than those in the heparin group (RR 0.32 (95% CI (0.22–0.47); *p* < 0.0001)). However, this difference did not translate into a difference in the transfusion requirements between the two groups (RR 1.02 (95% CI (0.93–1.12); *p* = 0.644). The fixed-effect model of trial sequential analysis was used, showing that citrate compared to heparin was more effective in decreasing the risk of bleeding. The cumulative Z-curve crosses the conventional boundary for benefit and the trial sequential monitoring boundary. (see Figure 4)

#### 3.4.5. HIT

Seven studies [17,19,20,21,22,24,25] found that the risk of HIT was low and did not significantly differ between the two groups (RR= 0.62 (95% CI (0.33–1.15); *p* = 0.126). The fixed-effect model of trial sequential analysis was utilized. A diversity-adjusted information size of 16,471 participants was calculated, founded on a HIT rate of 4.17% in the heparin group, relative risk reduction of 20%, alpha = 5% (two-sided), beta = 20%, I^2^ = 0%. The cumulative Z-curve crosses the conventional boundary for benefit. (see Figure 4)

### 3.5. Efficacy

#### Filter Lifespan

**Figure 5:**
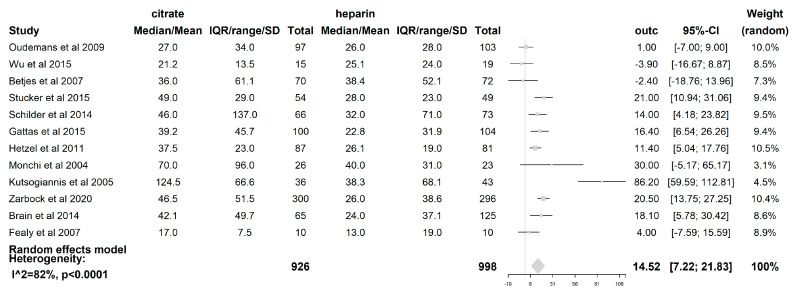
Forest plot of filter lifespan. Due to heterogeneity (*p* < 0.0001, I^2^ = 81.6%, H^2^ = 5.42), random effects model for filter lifespan was used. A significant difference of 14.52 h with 95% CI [7.22,21.83]; *p* < 0.0001 was found between citrate and heparin.

The filter lifespan was reported in all 12 included studies. The median filter lifespan was 14.52 h (95% CI (7.22–21.83); *p* < 0.0001), which was significantly longer when citrate was used than when heparin was used. (see Figure 5) The impact of the CRRT modality itself on the filter lifespan was also examined. In those studies where the CVVH [19,21,23,24,25,27,28] was used (621 patients), the difference in median filter life between citrate and heparin (5.89 h (95% CI (−0.05–11.84); *p* = 0.052)) was not statistically significant. However, with the use of the CVVHDF mode [18,22,26] (comprising 163 patients), a significant difference in median filter lifespan of 38.4 h (95% CI (10.54–66.26); *p* = 0.007) longer was seen when citrate was used compared to heparin. The median filter life is significantly longer for citrate when the modality CVVHDF is used compared to CVVH (*p* = 0.025). The dilution mode was shown to have an impact on FLS, with a median filter life 16.76 h longer (95% CI (4.43–29.09); *p* = 0.008)) when citrate was used compared to heparin in the predilution mode [18,19,20,21,24,27]. Additionally, the median filter life was also longer in the postdilution [22,23,25,26,28] mode when citrate was used (11.36 h (95% CI (0.13–22.59); *p* = 0.047). However, there was no difference in the filter lifespan between both substitution modes (*p* = 0.526). The risk of premature clotting was significantly lower when citrate was used (RR 0.44 (95% CI (0.29–0.67); *p* = 0.0001).

TSA with the DL approach and the (SJ) approach shows that the cumulative Z-curve crosses the conventional boundary for benefit and the sequential monitoring boundary in the DL approach, however, hits the trial sequential monitoring boundary in the (SJ) approach. The CVVHDF modality showed that in the DL approach, the cumulative Z-curve crosses the conventional boundary and the trial sequential monitoring boundary for benefit. This was not the case for the CVVH modality. The predilution subgroup showed that the cumulative Z-curve crosses the conventional boundary and the trial sequential monitoring boundary. This could not be demonstrated for the postdilution subgroup. (see Figures S3–S5; supplementary file).

### 3.6. Secondary Outcomes

There were no significant differences between the groups in 28-day mortality (RR= 1.08 (95% CI (0.89–1.31); *p* = 0.424), 90-day mortality (RR= 0.9 (95% CI (0.8–1.02); *p* = 0.110); renal recovery (*p* = 0.176) or requirement of renal replacement therapy (*p* = 0.816).

For the 3-months mortality, the fixed-effect model was used for TSA, and the results showed that the cumulative Z-curve crosses the futility boundary and enters the futility area. (see Figure S6; supplementary file)

### 3.7. Publication Bias

We assessed the potential publication bias for acid–base disturbances (metabolic acidosis: *p* = 0.652 for the Begg test and *p* = 0.928 for the Egger test, and metabolic alkalosis: *p* = 0.805 for the Begg test and *p* = 0.061 for the Egger test) and circuit life span (*p* = 0.583 for the Begg test and *p* = 0.410 for the Egger test). No potential publication bias was observed among the included trials.

## 4. Discussion

The present meta-analysis compared the safety and efficacy of citrate and heparin anticoagulation in CRRT across 12 RCTs involving 1592 patients. Overall, compared with heparin anticoagulation, we found that RCA in critically ill patients yielded a longer filter life and a lower risk of premature clotting of the extracorporeal circuit. Subgroup analysis showed a prolonged circuit life in the CVVHDF group and in both dilution groups. On the other hand, there was no statistically significant difference in acid–base disturbances between these two anticoagulation regimes. Not surprisingly, citrate anticoagulation was associated with an increased risk of hypocalcaemia, although there were no severe hypocalcaemia-related complications.

The findings indicate that if the protocol is strictly followed, metabolic derangement can be easily identified and controlled, without causing catastrophic clinical consequences. RCA reduced the risk of bleeding compared to heparin, but did not have an effect on transfusion requirements. Heparin did not lead to a higher risk of HIT. None of the anticoagulation strategies showed superiority in terms of renal recovery or mortality.

### 4.1. Safety Outcomes: Electrolyte Derangements and Acid–Base Disorders

Citrate administration causes strict regional anticoagulation by chelating calcium, and the resulting hypocalcaemia then causes inhibition of the coagulation cascade [29]. To interrupt the coagulation pathway, the target is to attain an iCa^++^ concentration below 0.4 mmol/L in the filter [29]. Anticoagulation can be achieved by a fixed citrate dose proportional to the blood flow, aiming for a citrate concentration of 3 mmol/L or titrating the citrate dose by measuring postfilter iCa^++^. The extracorporeal clearance of the calcium–citrate complex by convection or diffusion is the same as that of urea (sieving coefficient 0.87–1.0). During RCA-CRRT, approximately 30–70% of the calcium–citrate complex is removed by haemofiltration or moves across the membrane by diffusion during dialysis, and is lost in the ultrafiltrate or dialysate [6,30]. The remaining calcium–citrate complex reaching the systemic circulation is metabolized to bicarbonate by the mitochondrial citric acid cycle in the liver, kidney, and skeletal muscle [31]. The buffering effect of sodium citrate is proportional to the sodium ions it contains. One mole of trisodium citrate yields three moles of sodium bicarbonate. Extracorporeal losses of calcium require compensation by an exogenous infusion. Therefore, a systemic calcium infusion is necessary to replace the calcium lost with citrate [29]. When hypocalcaemia is severe, it can be life threatening, and patients may experience bronchospasm or laryngospasm, localized or generalized seizures, myocardial dysfunction and death [32]. Although RCA was associated with more hypocalcaemia events, no significant hypocalcaemia-related consequences were reported. Moreover, the ionized calcium level can easily be controlled.

Metabolic complexity is a major concern when performing RCA-CRRT. Three clinically important acid–base disorders are associated with RCA [33]. First, an accidental excessive citrate load may overwhelm the metabolizing capacity of a normal liver, resulting in acute, transient, yet life-threatening citrate intoxication and metabolic acidosis. Our meta-analysis did not show a higher incidence of metabolic acidosis in the citrate group, indicating that with enhanced monitoring, the occurrence of this feared complication can be avoided. Second, insufficient citrate metabolism in patients with severe liver failure, hypoxemia, severe lactic acidosis, and shock, is at risk of accumulation. In the setting of severe liver dysfunction, citrate clearance is impaired by approximately 50%, which means that liver failure patients are more susceptible to citrate accumulation and well-known complications associated with citrate toxicity. Indirect markers of citrate accumulation are a rising anion gap, worsening metabolic acidosis, and refractory ionized hypocalcaemia during increasing calcium infusion supplementation, which are all in favour of citrate accumulation. Detection can be performed by taking the calcium gap into account (i.e., the ratio between total and ionized calcium above 2.25 [34]). Citrate toxicity can be corrected by decreasing the citrate infusion rate, decreasing the blood pump speed and increasing the dialysate flow rate [35,36,37]. Although patients with liver failure were excluded from the RCTs in this meta-analysis, several studies have reported that RCA can be safely used in patients with liver failure [38]. Khadzhynov et al. reported that the incidence of citrate accumulation was low, occurring in only 2.99% of the 1070 patients treated with RCA-CVVHD. Moreover, it only occurs in patients with severe lactic acidosis due to multiorgan failure [39]. This was also confirmed in another study, where hyperlactatemia (>4 mmol/L) was strongly associated with citrate accumulation, indicating that the appearance of citrate accumulation is simultaneous with deteriorating multiorgan dysfunction and impaired organic substrate metabolism, and not only liver impairment, as previously understood [40]. In our present systematic review, the rate of citrate accumulation was low. Finally, metabolic alkalosis as a result of unintended citrate over-infusion, or decreased removal in the case of a decline in membrane performance, can occur. In CRRT-RCA, different citrate solutions are available for infusion. These citrate solutions are either hypertonic due to high sodium concentration or infused as a separate trisodium citrate solution or isotonic citrate solution due to a near physiologic sodium concentration administered as a calcium-free predilution replacement solution [34]. Hypertonic citrate formulations require hyponatremic replacement or dialysate solutions with reduced bicarbonate concentrations to prevent the development of electrolyte abnormalities [4]. In two studies [18,25], hypertonic citrate formulations were used, three other studies [21,23,28] used trisodium citrate solutions, and in all the other studies [17,19,20,22,24,26,27], isotonic solutions were applied. However, the hypernatremic events and incidence of metabolic alkalosis were trivial and not different between the two anticoagulation regimens.

### 4.2. Limitations

A limitation of our findings is that acid–base was measured only by pH and not concomitantly calculated by the apparent strong ion difference (SIDa) according to the simplified Stewart equation ([Na+] + [K+] + [Mg 2+] + [Ca 2+]) − ([Cl−] + [lactate−]) [41]. Naka et al. showed that a low dose of RCA (11 mmol/L) induced mild acidosis secondary to an increased strong ion gap (SIG) and decreased SIDa, which was fully self-corrected at the cessation of therapy [42]. In contrast, Egi et al. compared an RCA 14 mmol/L with an RCA 11 mmol/L solution, and found more metabolic alkalosis in the RCA 14 mmol/L arm. The daily infused citrate was higher in patients receiving RCA 14 mmol/L, which translated into a corresponding additional bicarbonate load that shifted the acid–base balance towards metabolic alkalosis [43]. Jacobs et al. also compared two diluted citrate solutions in patients treated with CVVH [44]. None of the Prismocitrate 10/2 patients reached a pH > 7.5, but 25% had an SIDa > 45 mmol/L. In the Prismocitrate-18 group, 10% of the patients had pH values > 7.5, whereas 93% were diagnosed with an SIDa > 45 mmol/L. Another limitation in assessing metabolic complications of citrate anticoagulation is the duration of CRRT, because in prolonged CRRT, high cumulative doses of citrate are administered. However, most trials examined only the first filter.

### 4.3. Safety Outcomes: Bleeding and Hit

Anticoagulation is mandatory to maintain the patency of the filter and prevent premature clotting, leading to downtime associated with an inadequate dose delivery, unintended blood loss, and increased costs [45,46]. However, a major drawback of anticoagulation is its association with significant adverse events, such as an increased risk of bleeding or unintended metabolic derangements [47]. Heparin and regional citrate are the most frequently used pharmacological products to obtain adequate anticoagulation. Heparin is widely available at a low cost and with a short half-life. Its action can be simply antagonized by administering protamine [48]. Although monitoring is possible with routine tests, the activated partial thromboplastin time, aPTT itself, is an unreliable predictor of bleeding, and its half-life can be increased in renal failure. Other disadvantages include heparin resistance and heparin-induced thrombocytopenia (HIT) [48,49,50,51]. Low-molecular-weight heparins (LMWH) have been investigated as an alternative for heparin, but even though weight-based dosing is possible and no monitoring is needed, there is a risk of accumulation in kidney failure. The incidence of HIT is lower when using LMWH compared to unfractionated heparin. Administration of protamine induces only an incomplete reversal of action. Monitoring requires nonroutine tests with anti-Xa assays, which are expensive and do not reliably predict bleeding and antithrombotic efficacy [52]. Our meta-analysis showed that bleeding complications in patients randomised to the citrate group were significantly lower compared with the heparin group. However, this was not translated to the transfusion requirements between the two groups. The incidence of HIT was low and not different between the groups.

### 4.4. Filter Lifespan Efficacy

This meta-analysis demonstrated a significantly longer circuit survival for treatments performed with citrate compared with heparin during CRRT. There is a belief that with the use of convection-based CVVH, more large molecules will be removed compared with diffusion-based CVVHD, but at the expense of a shorter filter life [53]. Haemofiltration requires higher blood flows for the same CRRT dose, leading to more flow limitation and more frequent stasis of blood flow. CVVHDF combines haemofiltration with haemodialysis with less haemoconcentration at relatively lower blood flows, and may thus increase circuit survival. Subgroup analysis regarding the mode of therapy demonstrated that filter life is significantly longer when CVVHDF is used compared to CVVH with use of citrate anticoagulation compared to heparin.

In predilution CRRT, substitution fluids are administered before the filter, thus diluting the blood in the filter, decreasing haemoconcentration, and improving rheological conditions [54]. It has also been suggested that with predilution, the membrane performance is better due to reduced clogging, which is the deposition of proteins and red cells on the membrane. The impact of the dilution mode revealed that FLS was superior in the predilution, as well as in the postdilution mode, when citrate was used, with no effect on filter life between pre- and postdilution. However, circuit patency and filter lifespan are affected not only by anticoagulant drugs, modality of CRRT and the administration of substitution, but also by the access catheter, blood flow and filtration fraction [55]. The risk of premature clotting was significantly lower when citrate was used.

### 4.5. Secondary Outcomes: Mortality and Renal Recovery

The prevalence of AKI in critically ill patients is high, as it occurs in more than half of ICU patients and is associated with increased morbidity and mortality [56]. CRRT has improved significantly since its first implementation in 1977, and has become the modality of choice in the ICU due to its superiority in haemodynamically unstable patients compared to IHD [57,58]. Recent results from a meta-analysis suggest that CRRT may increase renal recovery compared with intermittent haemodialysis (IHD), nevertheless failing to demonstrate a difference in mortality between the different modalities [59]. Our meta-analysis showed no difference in mortality between the two types of anticoagulation. However, two of the included studies in our meta-analysis (Zarbock et al. [17] and Schilder et al. [21]) were terminated early, and were therefore underpowered to reach definitive conclusions about the comparative effect of these anticoagulation strategies on mortality. Meta-analysis could not demonstrate an impact of anticoagulation on renal recovery. However, Oudemans et al. [25] showed a higher rate of renal recovery in patients randomised to citrate, particularly in surgical patients, patients with sepsis and severe organ failure, and in relatively younger patients. Schilder et al. [21] failed to show this independency of RRT, which could be explained by the more ischaemic aetiology of AKI, which may be attributed to a worse renal prognosis. There are several limitations in the RCTs performed, explaining why this meta-analysis failed to demonstrate an effect of anticoagulation on mortality and kidney recovery. First, all the trials had a high risk of bias due to blinding. Second, a major confounding factor in all the trials is the heterogeneity of the participants in regard to age, percent of patients with sepsis, percent who were surgical as opposed to medical ICU patients, severity of illness, pre-existing renal function, haemodynamic instability, and the accompanying use of vasoactive agents. Third, the modality and dose of RRT and the use of cointerventions were also limitations.

### 4.6. Limitations

Several limitations may be recognized in our present study. First, most studies are single-centre studies with a small sample size, and are thus underpowered to reveal differences in mortality. Second, the heterogeneity was high among the included studies. Various modalities of CRRT and RCA protocols were utilized using different citrate concentrations. Some studies adjusted the citrate dose based on postfilter iCa^++^, whereas others applied a fixed dose. The applied anticoagulation strategy in the control arm also differed between the studies. This can affect circuit patency and filter lifespan. In addition, vascular access and patient factors are nearly as important as anticoagulation methods in affecting filter survival. Moreover, there is a wide variation in nursing expertise to trouble shoot and approach alarms. Third, patients with liver failure were excluded, and citrate may be associated with a higher level of metabolic complications due to lower hepatic clearance. Additionally, the enrolled patients were not at a high risk of bleeding. Additionally, there was no uniform definition or grading for bleeding events, so potential bias might be introduced into the safety results. Fourth, blinding of the investigators or the treating intensivist was not possible because of the nature of the interventions, which could introduce potential performance and detection bias. Finally, a considerable amount of follow-up data were missing, which may result in an ascertainment bias.

Prior meta-analyses have been published assessing the safety and efficacy of regional citrate anticoagulation versus heparin anticoagulation [60,61]. However, the present systematic review and meta-analysis was registered in PROSPERO and reported according to the Preferred Reporting Items for Systematic Reviews and Meta-analyses (PRISMA) [12]. Additionally, we performed TSA to provide more accurate, precise, and unbiased information to clinicians to assess the conclusiveness of this meta-analysis.

## 5. Conclusions

Our meta-analysis found that neither the incidence nor the magnitude of metabolic derangements were of concern when using regional citrate anticoagulation for CRRT. Citrate-related hypocalcaemia was observed more frequently when citrate was used, but no severe adverse events related to this electrolyte disturbance were reported. Citrate anticoagulation for CRRT is superior to heparin anticoagulation in prolonging circuit life, and the incidence of bleeding events is consistently lower. However, an impact of citrate on mortality could not be demonstrated, and citrate can be recommended as the anticoagulant of choice during CRRT in critically ill patients. Due to its increased complexity, an appropriate protocol and careful monitoring are needed, and well-designed studies should be conducted to explore the appearance of citrate accumulation in deteriorating multi-organ dysfunction, and to clarify the impact of citrate on survival as well.

## Figures and Tables

**Table 1:** Main characteristics of the studies and participants.

Study	Study Design	Follow-up	Inclusion Criteria	Exclusion Criteria	Age (Years) Mean ± SD, Median (Range)	Sex (M/F)	Endpoints
Betjes et al. [23]; 2007	Parallel RCT	NR	AKI requiring RRT	Contraindication anticoagulant; patients with anticoagulation for medical reason	H: 55.2 ± 2.8 C: 57.8 ± 4.2	H: 19/8 C: 15/6	AE, Circuit survival, bleeding
Schilder et al. [21]; 2014	Parallel RCT; multicenter	90 days	AKI requiring RRT	Risk of bleeding; <18 or >80 y; need for systemic anticoagulation or HIT; chronic RT, administration of activated protein C or PE therapy	C: 67 (36–87) H: 67 (23–85)	C: 44/22 H: 49/24	Mortality, renal outcome, safety and efficacy
Fealy et al. [27]; 2007	Cross-over RCT	NR	AKI; RIFLE “F”, requiring CRRT	Liver failure; suspected ischemic hepatitis; contraindication heparin/protamine	70.5 (63.4–76.5)	9/1	Circuit clotting, bleeding
Brain et al. [26]; 2014	Parallel RCT	90 days	AKI requiring RRT	Weight < 30 kg; contraindication anticoagulant, chronic RT; pregnancy/lactation; therapeutic hypothermia; previous participation in study; indication different dialysis prescription	C: 64 ± 13 H: 51 ± 17	C: 12/7 H: 7/4	Safety and efficacy, RT dependence, mortality
Gattas et al. [20]; 2015	Parallel RCT; multicenter	NR	AKI requiring RRT, no contraindication for anticoagulation of CRRT circuit, IC	Stay ICU < 24 h; < 18 y; pregnant/lactation; liver failure; allergy to heparin/protamine; HIT; chronic dialysis	C: 66.4 ± 14.3 H: 66.8 ± 14.9	C: (74/105) H: (72/107)	FLS, mortality, cytokines change, transfusion, LOS duration of CRRT,
Hetzel et al. [19]; 2011	Parallel RCT; multicenter	Discharge from ICU up to 30 d	AKI requiring RRT; MV	HIT; need of systemic UFH; pH > 7.50 and BE > +4 mmol/L; pregnancy/lactation; chronic RT; participation another study <3 m; previous participation in study	C: 61.7 ± 15.3 H: 65.1 ± 12.5	C: 57/30 H: 59/24	Efficacy and safety
Kutsogiannis et al. [18]; 2005	Parallel RCT; multicenter	Hospital discharge or death	AKI	Contraindication to anticoagulant or requirement of systemic heparin for medical reasons; pregnancy	C: 66.5 ± 14.5 H: 63.9 ± 21.2	C: 7/16 H: 8/14	FLS and bleeding
Monchi et al. [28]; 2004	Cross-over RCT	NR	AKI	Cirrhosis; severe coagulopathy; high risk of bleeding; allergy to heparin	H: 64 (52–74) C: 67 (52–77)	H: 11/12 C: 12/14	Efficacy and safety
Oudemans et al. [25]; 2009	Parallel RCT	90 days	AKI requiring RRT	Cirrhosis Child–Pugh C; bleeding; surgery within 24 h before CVVH; need of therapeutic anticoagulation; HIT; chronic RT; DNR	C: 73 (64–79) H: 73 (67–79)	C:(66/31) H: (70/33)	AE, transfusion, metabolic, and clinical outcomes FLS
Stucker et al. [22]; 2015	Parallel RCT	90 days	AKI requiring RRT according to RIFLE	Active hemorrhagic disorders or PC < 50 × 109/L; HIT; severe liver failure (=factor V < 20%); on waiting list for liver transplantation	C: 60 ± 14 H: 65 ± 16	C: 32/22 H: 32/17	FLS, AE, RRT dose, survival, LOS
Wu et al. [24]; 2015	Parallel RCT	NR	AKI requiring CVVH for >48 h; no contraindication for anticoagulation	Aged ≤ 18 years; coagulopathy; anticoagulation/hemostatic agent <24 h prior to enrolment; need anticoagulation/hemostatic agent for other reasons	C: 48.1 ± 3.9 LMWH: 45.2 ± 4.1	C: 10/5 LMWH: 12/7	FLS, premature clotting, AE
Zarbock et al. [17]; 2020	Parallel RCT	90 days	AKI KDIGO 3 requiring RRT + sepsis/septic shock, vasopressor or refractory fluid overload, 18–90 years, intention to provide full treatment for at least 3 days	Bleeding risk; diseases with hemorrhagic diathesis; chronic RT; need of therapeutic anticoagulation; HIT; allergy to anticoagulants; AKI due to occlusion/lesion renal arteries; AKI due to interstitial or glomerulonephritis, vasculitis or urinary tract obstruction; DNR; HUS/TTP; persistent lactate acidosis due to ALF/shock; kidney transplant <12 months; pregnancy/lactating; no CRRT machine free; participation in another trial <3 months; persons with dependency on sponsor/investigator; detained person	C: 67.5 ± 12.3 H: 67.6 ± 12.5	C: (206/94) H: (207/89)	FLS and 90-day mortality, bleeding, and new infections

AE: adverse events; AKI: acute kidney injury; ALF: acute liver failure; aPTT: activated partial thromboplastin time; AST: aspartate aminotransferase; BE: base excess; BUN: blood urea nitrogen; C: Citrate; CI: contraindication; CKD: chronic kidney disease; CRRT: continuous renal replacement therapy; d: days; DIC: diffuse intravascular coagulopathy; DNR: do-not-resuscitate order; FLS: filter life span; GI: gastro-intestinal; H: heparin; h: hours; Hb: haemoglobin; HIT: heparin-induced thrombocytopenia; HUS: haemolytic uremic syndrome; iCa^++^: ionized calcium; ICU: intensive care unit; INR: international normalised ratio; KDIGO: Kidney Disease Improving Global Outcomes; LMWH: low-molecular-weight heparin; IC: informed consent; LOS: length of stay; MV: mechanical ventilation; MOF: multiple organ failure; NR: not reported; PC: platelet count; RCT: randomised controlled trial; RIFLE: risk, injury, failure, loss and end-stage kidney disease; RRT: renal replacement therapy; Scr: serum creatinine; TTP: thrombotic thrombocytopenic purpura; UFH: unfractionated heparin; UO: urine output.

**Table 2:** Protocols of RCA-CRRT.

Author	Study Participants	Other Anticoagulant	Citrate Anticoagulation	Replacement Fluid	Dialysate	Additives
Oudemans-van Straaten et al. [25]	RCA: 97 Nadroparin: 103	IV bolus of 2850 IU nadroparin, or 3800 IU if > 100 kg, followed by 380IU/h or 456 IU/h, respectively, no monitoring of anti-Xa	Hospital pharmacy prepared containing 500 mmol/L citrate, 1352 mmol/L sodium, and 148 mmol/L hydrogen	C: SH 44 HEP or SH 53 HEPnadroparin: -if metabolic acidosis and/or hyperlactatemia: SH 53 HEP -if no acidosis and lac < 5 mmol/L: BH504		For iCa^++^: 0.9–1.0 mmol/L: calcium-magnesium-chloride (0–0.4mmol/h of calcium and 0–0.24 mmol/h magnesium)
Wu et al. [24]	RCA: 15 Dalteparin: 19	Dalteparin loading dose: 40 IU/kg; maintenance: 4 IU/kg per h	Citrate- (7 mmol/L) and bicarbonate (17 mmol/L)-based calcium-free replacement fluid	Bicarbonate-based		Calcium and magnesium supplement
Betjes et al. [23]	RCA: 21 Hep: 27	Bolus of 3000–5000 IU, followed by continuous infusion 1500 IU/h~APTT 50–70 s	Trisodium citrate solution (13%) at 55 mL/min	H: HF32bic C: SH 44 HEP		Calcium chloride if iCa^++^ < 0.9 mmol/L
Stucker et al. [22]	RCA: 54 Hep: 49	Unfractioned heparin 500 IU/h	Prismocitrate 18	Prismasol	Prismocal B22	
Schilder et al. [21]	RCA: 66 Hep: 73	Heparin bolus of 5000 IU and 833 IU/h~APTT 50 s	HFCitPre (citrate 39.9 and sodium 139.9) mmol/L	H: HF32bic or BH504		Calciumgluconate for iCa^++^: 1–1.35 mmol/L
Fealy et al. [27]	10	H: 1500 u/h before hemofilter;Protamine 15 mg/h after hemofilter	Citrate-buffered RF: infused prefilter at 28 mmol/h	H: lactate buffered RF prefilter at 2000 mL/h. If lactate > 5 mmol/L: lactate-free bicarbonate-buffered RFC: citrate buffered RF prefilter at 2000 mL/h		Calcium: 4mmol/h magnesium: 2 mmol/h
Gattas et al. [20]	RCA: 105 Hep: 107	1000–1500 IU/h regional UFH plus 10–15 mg/h protamine	Prismocitrate 10/2	C: Hemosol B0 postfilter at 200 mL/h H: Hemosol B0 prefilter at 2000 mL/h; postfilter at 200 ml/h	C: Prismocal at 500 mL/h H: Hemosol B0 at 500 mL/h	Calcium chloride for iCa^++^: 1–1.2 mmol/L
Hetzel et al. [19]	RCA: 87 Hep: 83	Systemic heparin	HF-citrate 13 mmol/L and 140 mmol/L sodium	H: HF-bicarbonate prefilter at 42 ml/kg/h		Calcium chloride
Monchi et al. [28]	Hep:12 RCA: 8	Bolus of 2000–5000 IU of heparin into the circuit + continuous infusion of 1000 U/h~APTT 60–80 s	Trisodium citrate 1 mmol/ml	Postfilter RF: (Na^+^ 120, K^+^ 1, Cl^−^ 122, Mg^2+^ 0.5) mmol/L H: + 25–28 mmol/L Nabic + 1.1 mmol/L calciumchloride		Calcium chloride for iCa^++^: 1.0–1.15 mmol/L Magnesiumsulfate 1 mmol
Kutsogiannis et al. [18]	RCA: 16 Hep: 14	Heparin bolus: 50 IU/kg for~APTT ≤ 35 s, followed by protocolized algorithm for~APTT 45–65 s	Tricitrisol at 190 mL/h (25 mmol/h)	RF: Na^+^ 117, Mg^2+^ 0.70, Cl^−^ 117) mmol/L+ HCO^3−^: 33.3–50 mmol/L; at 1000 mL/h prefilter	Solution: Na^+^ 117, Mg^2+^ 0.70, Cl^−^ 117) mmol/LIn case of Hep: Solution+ HCO^3−^: 33.3–50 mmol/L; at 1000 mL/h	Calcium chloride
Zarbock et al. [17]	RCA: 300 Hep: 296	Heparin for~APTT 45–60 s	4% sodiumcitrate Prismocitrate 10/2 or 18/0 30% sodiumcitrate	-Multibic -Phoxilium -sodium chloride	-K2 or K4 -Prismocal -HDE 2/0	
Brain et al. [26]	RCA: 19 Hep: 11	Heparin 1000 IU/h;~APTT 50–80 s	Prismocitrate 18	0.9% normal saline	In case of citrate: Prismocal B22 In case heparin: Hemosol B0	Calcium chloride infusion if iCa^++^ < 1.1 mmol/L

APTT: activated partial thromboplastin time; iCa^++^: ionized calcium; Hep: heparin; RCA: regional citrate anticoagulation; RF: replacement fluid. Prismocitrate 10/2 (Gambro Hospal, Lund, Sweden); Prismocitrate 18 (Gambro Hospal, Lund, Sweden); HF32bic (Dirinco, The Netherlands); SH 44 HEP (Dirinco, The Netherlands); SH 53 HEP (Dirinco, Rosmalen The Netherlands), lactate-buffered RF (Hemofiltration Solutions, Baxter, Sydney, Australia), bicarbonate-buffered RF (Gambro Hospal, Sydney, Australia), Ca-containing RF: (Qingshan Likang, Pharmaceutical Co. Ltd., Chengdu, China), HFCitPre: (Dirinco, Oss, The Netherlands), Tricitrisol: (Citra Laboratories, Braintree, MA, USA).

**Table 3:** Characteristics of CRRT.

Study	Modality CRRT	Device	Blood Flow mL/min	Citrate Target (mmol/L) Blood Flow	Dilution Mode	Postfilter iCa^++^ Target (mmol/L)	CRRT Dose
Oudemans-van Straaten et al. [25]	CVVH	Diapact and Aquarius	220	3	Postdilution	NR	NR
Wu et al. [24]	CVVH	Aquarius and Multifiltrate	180–200	NR	Predilution	NR	4 L/h
Betjes et al. [23]	CVVH	Hygieia Ultima	150	NR	Postdilution	0.25–0.3	25 mL/h
Stucker et al. [22]	CVVHDF	Prismaflex	100–200	3	2/3 in Predilution mode &and 1/3 in postdilution	0.25–0.3	30 mL/kg/h
Schilder et al. [21]	CVVH	NR	180	NR	Predilution	Not measured	2000–4000 mL/h
Fealy et al. [27]	CVVH	Kimal Hygieia	150	NR	Predilution	NR	NR
Gattas et al. [20]	CVVH CVVHDF	Prismaflex and Aquarius	150–200	2.5–3	Predilution	Not measured	2000–4000 mL/h
Hetzel et al. [19]	CVVH	Multifiltrate	Blood flow to HF: 3/1	4	Predilution	<0.4	NR
Monchi et al. [28]	CVVH	Baxter CM11-CM14 device	150	4.3	Postdilution	<0.3	35 mL/kg/h
Kutsogiannis et al. [18]	CVVHDF	Prisma CFM System	125		Predilution	0.25–0.35	NR
Zarbock et al. [17]	CVVH CVVHD CVVHDF	Fresenius MC Prismaflex BBraun	100	NR	Pre- and postdilution	0.25–0.35	30 mL/kg/h
Brain et al. [26]	CVVHDF	Prismaflex	150–250 (according to weight)	3	Postdilution	Prefilter 0.3–0.44	NR

CVVH: continuous veno–venous hemofiltration; CVVHD; continuous veno–venous hemodialysis; CVVHDF: continuous veno–venous hemodiafiltration; Hep: heparin; NR: not reported; NA: no anticoagulation; RCA: regional citrate anticoagulation; Aquarius hemodialysis system (Baxter International Inc., Chicago, IL, USA); multifiltrate hemodialysis system (Fresenius Medical Care, Bad Homburg, Germany); Kimal Hygieia CRRT machine (Kimal, PLC, Uxbridge, UK); Prismaflex (Gambro, Lundia, Sweden).

## Data Availability

Data available in a publicly accessible repository. The data presented in this study are openly available and can be downloaded.

## Supplementary Materials

The following supporting information can be downloaded at: https://www.mdpi.com/article/10.3390/life13051198/s1.
