# Supplementary material for: Regional Citrate Anticoagulation in Continuous Renal Replacement Therapy: Is Metabolic Fear the Enemy of Logic? A Systematic Review and Meta-Analysis of Randomised Controlled Trials
Source: Life (Basel). 2023 May 17;13(5):1198. doi: 10.3390/life13051198 (PMC10221969; doi:10.3390/life13051198)
Supplement: Supplementary file 1 [file life-13-01198-s001.zip › life-2309957-supplementary.pdf]

Regional Citrate Anticoagulation in Continuous Renal Replacement Therapy: Is Metabolic Fear the Enemy of Logic? A Systematic Review and Meta-Analysis of Randomised Controlled Trials

Rita Jacobs [1,*], Walter Verbrugghe [1], Karolien Dams [1], Ella Roelant [2], Marie Madeleine Couttenye [3,4], Dirk Devroey [5] and Philippe Jorens [1,4]

Supplementary materials

<u>Table S1</u>**:** Comparisons in evidence according to GRADE: Citrate versus heparin anticoagulation CRRT

Certainty assessment						N of patients/events		Effect		Certainty
**N of studies**	**Risk of bias**	**Inconsistenty**	**Imprecision**	**Indirectness**	**Publication bias**	**Citrate**	**heparin**	**Relative (95% CI)**	**Absolute (95% CI)**	
12	serious	serious	Not serious	Not serious	Undectected	926	998	-	median **14.52 h longer** (7.22 to 21.83)	⊕⊕◯◯ Low
12	serious	Not serious	serious	Not serious	Undectected	35/815 (4.3%)	117/812 (14.4%)	**RR 0.32** (0.22 tot 0.47)	**98 less per 1.000** (from 112 less to 76 less)	⊕⊕◯◯ Low
9	serious	Not serious	serious	Not serious	Undectected	28/638 (4.4%)	7/631 (1.1%)	**RR 3.81** (1.67 tot 8.66)	**31 more per 1.000** (from 7 more to 85 more)	⊕⊕◯◯ Low
6	serious	Not serious	serious	Not serious	Undectected	122/347 (35.2%)	110/336 (32.7%)	**RR 1.08** (0.89 tot 1.31)	**26 more per 1.000** (from 36 less to 101 more)	⊕⊕◯◯ Low
7	serious	Not serious	serious	Not serious	Undectected	35/686 (5.1%)	20/683 (2.9%)	**RR 1.71** (0.99 tot 2.93)	**21 more per 1.000** (from 0 less to 57 more)	⊕⊕◯◯ Low
8	serious	Not serious	serious	Not serious	Undectected	25/599 (4.2%)	26/610 (4.3%)	**RR 1.46** (0.52 tot 4.11)	**20 more per 1.000** (from 20 less to 133 more)	⊕⊕◯◯ Low
6	serious	Not serious	serious	serious	Undectected	14/716 (2.0%)	17/727 (2.3%)	**RR 1.83** (0.40 tot 8.38)	**19 more per 1.000** (from 14 less to 173 more)	⊕◯◯◯ Very low
							0.0%		**0 minder per 1.000** (from 0 minder tot 0 minder)	
8	serious	Not serious	Not serious	Not serious	Undectected	338/704 (48.0%)	334/705 (47.4%)	**RR 1.02** (0.93 tot 1.12)	**9 more per 1.000** (from 33 less to 57 more)	⊕⊕⊕◯ Moderate
7	serious	Not serious	serious	Not serious	Undectected	17/727 (2.3%)	31/732 (4.2%)	**RR 0.62** (0.33 tot 1.15)	**16 less per 1.000** (from 28 less to 6 more)	⊕⊕◯◯ Low

CI: Confidence interval; **RR:** Risk ratio

<u>Figure S1</u> : Forest plots and TSA of electrolyte disorders

A

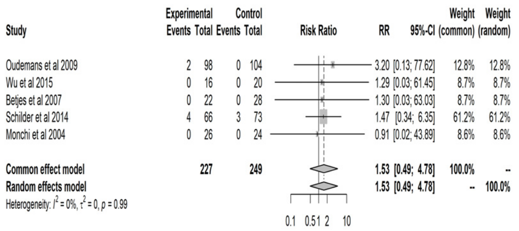

B

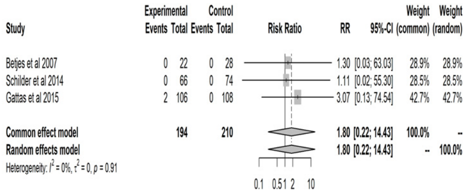

C

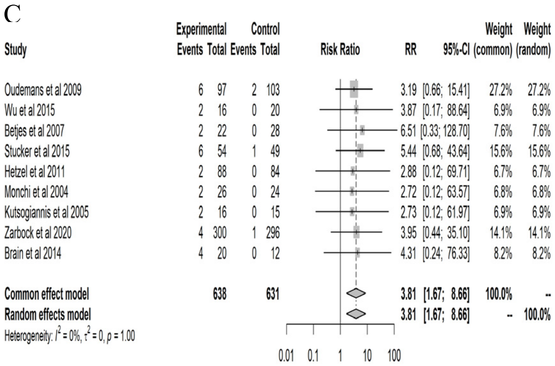

D

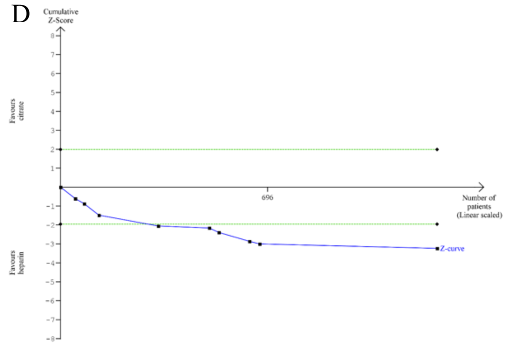

A: fixed effect model RR for hypernatremia for citrate versus heparin 1.53 [0.49,4.78]; p=0.467.

B: fixed effect model RR for hypercalcemia for citrate versus heparin 1.80 [0.22,14.43]; p=0.582).

C: fixed effect model: RR for hypocalcemia for citrate versus heparin 3.81 [1.67,8.66]; p=0.001.

D: fixed effect model model of trial sequential analysis for hypocalcemia. A diversity-adjusted information size of 64219 participants calculated on a hypocalcemia rate of 1.11% in the heparin group, relative risk reduction 20%, alpha=5% (two sided), beta=20%, I^2=0%. Boundary required information size is ignored due to too little information use (1.97%). The cumulative Z-curve stays crosses the conventional boundary for benefit.

<u>Figure S2</u> : Forest plot and TSA of citrate accumulation

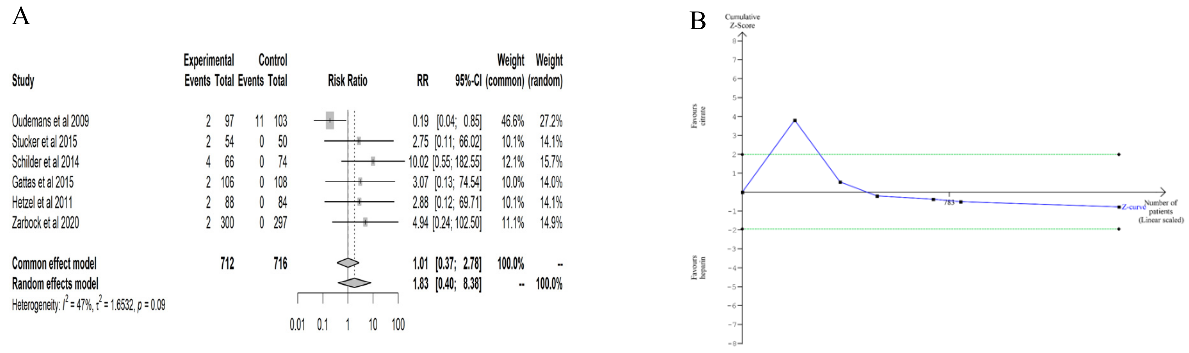

A: random effects model RR of citrate accumulation in citrate versus heparin 1.83 [0.40,8.38]; p=0.438.

B: random effects model of trial sequential analysis for citrate accumulation. A diversity-adjusted information size of 82586 participants calculated on a citrate accumulation rate of 1.89% in the heparin group, relative risk reduction 20%, alpha=5% (two sided), beta=20%, I^2=56.01%. Boundary required information size is ignored due to too little information use (1.73%).

Figure S3: funnel plot and TSA of filter lifespan

A

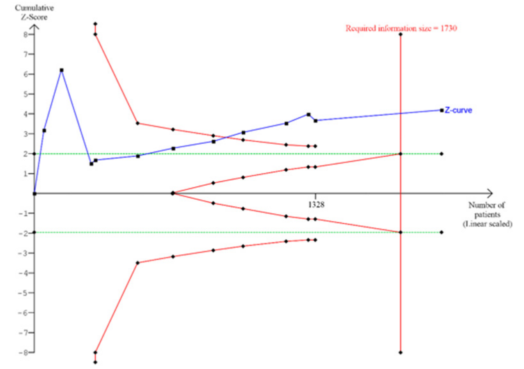

B

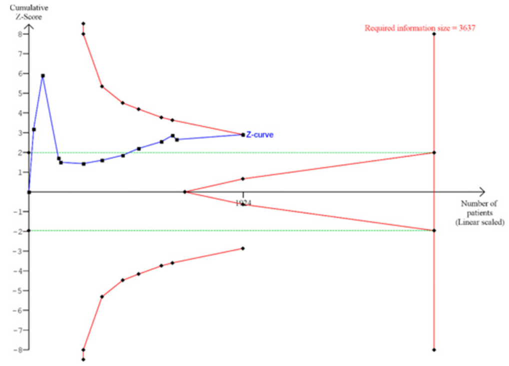

C

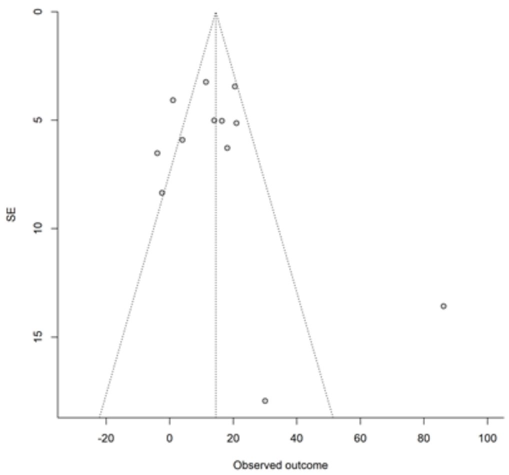

A: trial sequential analysis with the DL approach for filter lifespan. A diversity-adjusted information size of 1730 patients was calculated on a mean difference of 14.71, variance of 1616.87, I^2=86.42%, alpha=5% (two sided) and beta=20%. The cumulative Z-curve crosses the conventional boundary for benefit and the trial sequential monitoring boundary.

B: trial sequential analysis with the Sidik-Jonkman (SJ) approach for filter lifespan. A diversity-adjusted information size of 3637 patients was calculated based on a mean difference of 16.06, variance of 1616.87, I^2=94.58%, alpha=5% (two sided) and beta=20%. Two trials (Wu and Fealy) are ignored in interim looks due to too low information use (<1.0%). The cumulative Z-curve crosses the conventional boundary for benefit and hits the trial sequential monitoring boundary.

C: funnel plot of filter life span

Figure S4: forest plots and TSA of subgroup analysis for filter lifespan on modality of CRRT

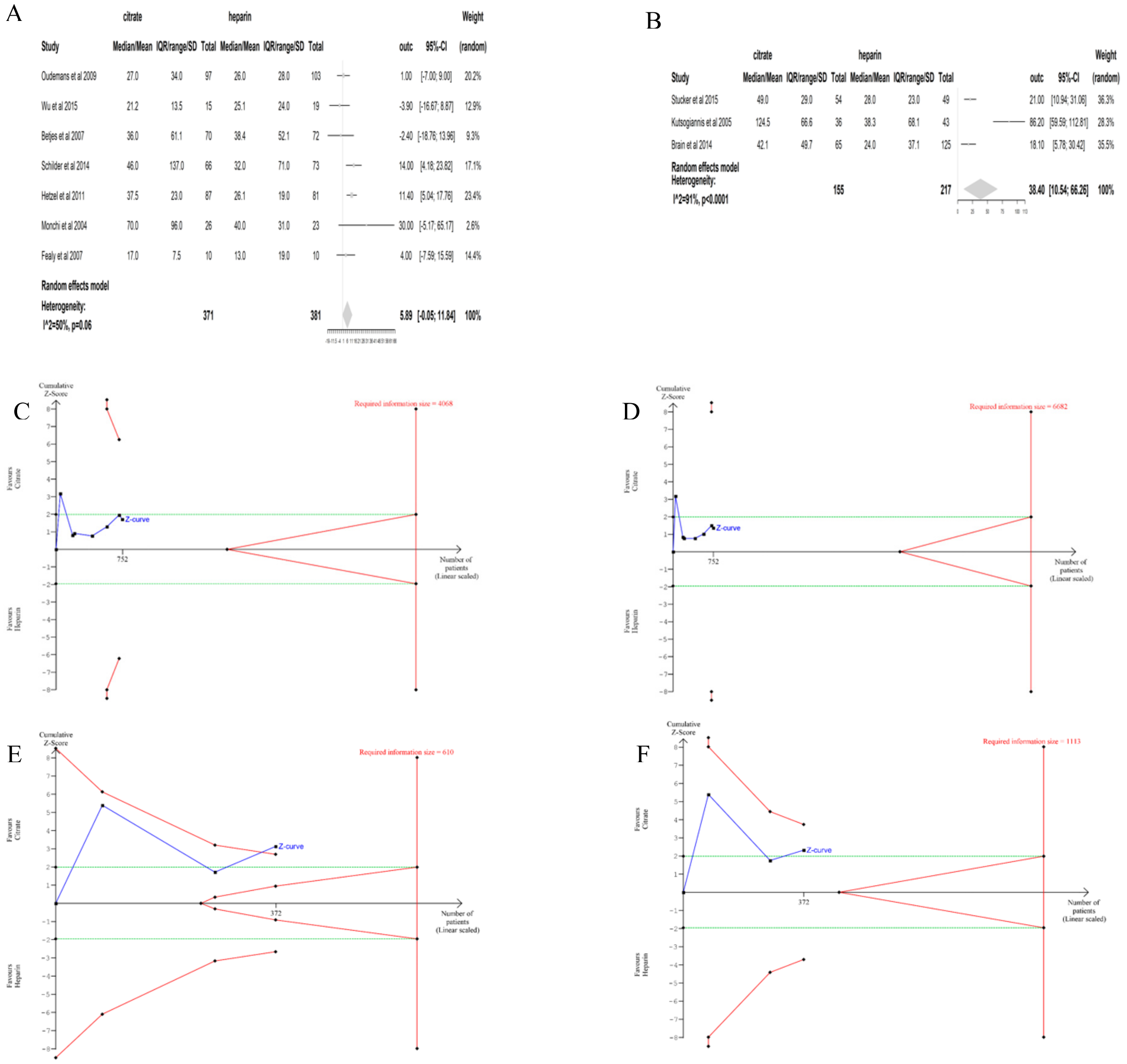

A: due to heterogeneity (p=0.06, I^2=49.6%, H^2=1.98 with the random effects model for the CVVH modality between citrate and heparin, we found no difference in median filter life of 5.89h with a 95% CI [-0.05,11.84]; p=0.052.

B: random effects model due to heterogeneity was use (p<0.0001, I^2=90.96%, H^2=11.07. A difference in median filter life between citrate and heparin for the CVVHDF modality was found of 38.4h with 95% CI [10.54,66.26];p=0.007.

C: trial sequential analysis with the DL approach for CVVH modality. A diversity-adjusted information size of 4068 patients was calculated based on a mean difference of 8.05, variance of 1151.66, I^2=86.29%, alpha=5% (two sided) and beta=20%. Two trials (Wu and Fealy) are ignored in interim looks due to too low information use (<1.0%).

D: trial sequential analysis with the SJ approach for CVVH modality. A diversity-adjusted information size of 6682 patients was calculated based on a mean difference of 8.86, variance of 1151.66, I^2=93.1%, alpha=5% (two sided) and beta=20%. Three trials (Monchi, Wu and Fealy) are ignored in interim looks due to too low information use (<1.0%). The cumulative Z-curve does not cross the conventional boundary or the trial sequential monitoring boundary.

E: trial sequential analysis with the DL approach for CVVHDF modality. A diversity-adjusted information size of 610 patients was calculated based on a mean difference of 30.49, variance of 2298.45, I^2=87.23%, alpha=5% (two sided) and beta=20%. The cumulative Z-curve crosses the conventional boundary and the trial sequential monitoring boundary.

F: Trial sequential analysis with the SJ approach for CVVHDF modality. A diversity-adjusted information size of 1113 patients was calculated because of a mean difference of 31.97, variance of 2298.45, I^2=93.62%, alpha=5% (two sided) and beta=20%. The cumulative Z-curve crosses the conventional boundary but not the trial sequential monitoring boundary.

Figure S5: forest plots and TSA of subgroup analysis- dilution mode

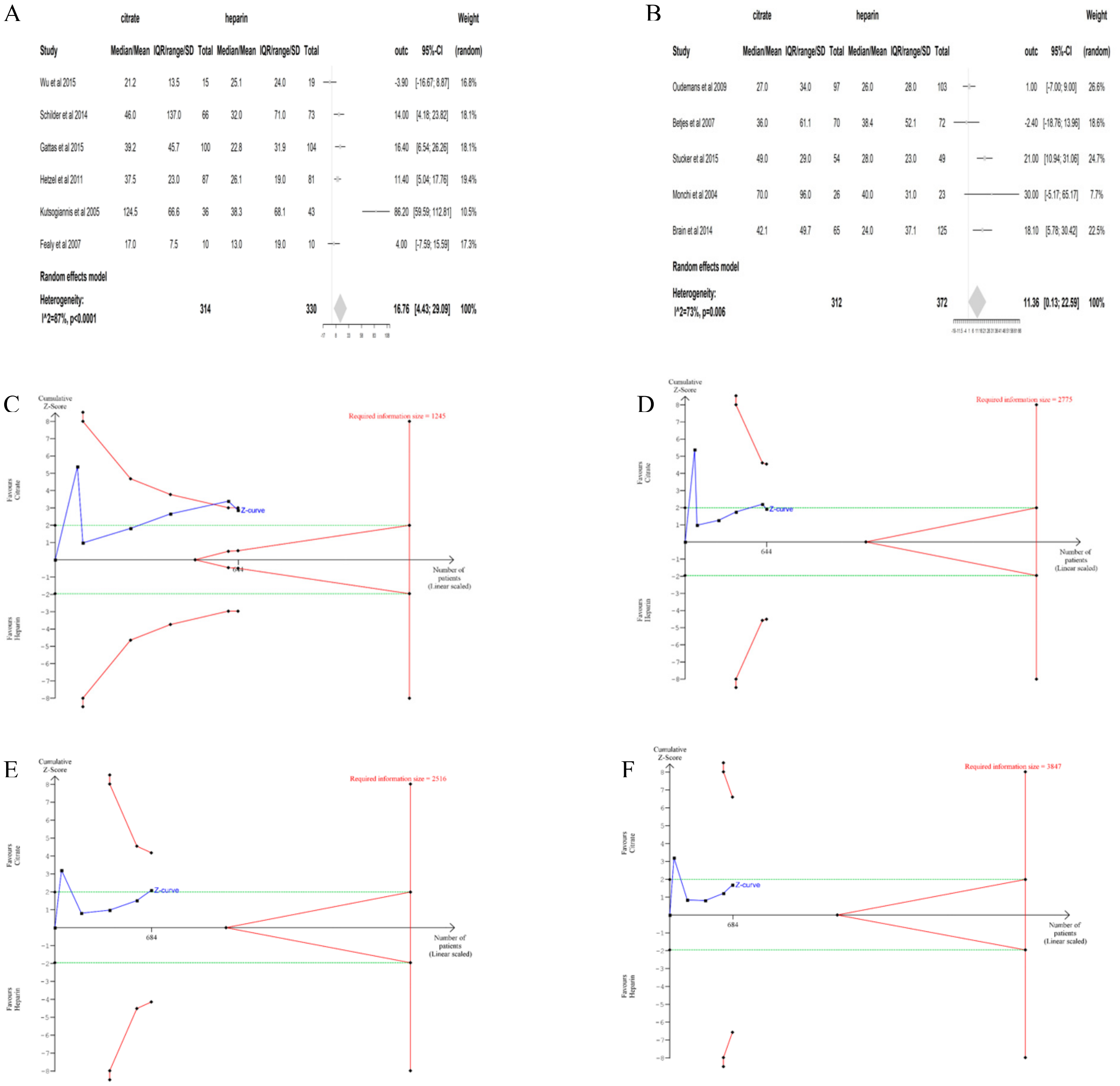

A: random effects model for predilution subgroup was used due to heterogeneity (p<0.0001, I^2=87.1%, H^2=7.7 a significant difference in median filter life was found between citrate and heparin: 16.76h with a 95% CI [4.43,29.09]; p=0.008.

B: due to heterogeneity (p=0.006, I^2=72.5%, H^2=3.6 with the random effects model a difference in median filter life for the postdilution subgroup of 11.36h with a 95% CI [0.13,22.59]; p=0.047 between citrate and heparin.

C: trial sequential analysis with the DL approach for predilution subgroup. A diversity-adjusted information size of 1245 patients was calculated based on a mean difference of 16.55, variance of 1160.09, I^2=89.24%, alpha=5% (two sided) and beta=20%. The cumulative Z-curve crosses the conventional boundary and the trial sequential monitoring boundary.

D: trial sequential analysis with the SJ approach for predilution subgroup. A diversity-adjusted information size of 2775 patients was calculated based on a mean difference of 17.80, variance of 1160.09, I^2=95.86%, alpha=5% (two sided) and beta=20%. One trial (Fealy) is ignored in interim looks due to too low information use (<1.0%). The cumulative Z-curve crosses the conventional boundary but not the trial sequential monitoring boundary.

E: trial sequential analysis with the DL approach for postdilution subgroup. A diversity-adjusted information size of 2516 patients was calculated based on a mean difference of 13.20, variance of 1823.71, I^2=86.92%, alpha=5% (two sided) and

beta=20%. The cumulative Z-curve hits the conventional boundary and does not cross the trial sequential monitoring boundary.

F: trial sequential analysis with the SJ approach for postdilution subgroup. A diversity-adjusted information size of 3847 patients was calculated based on a mean difference of 14.36, variance of 1823.71, I^2=92.77%, alpha=5% (two sided) and beta=20%. The cumulative Z-curve does not cross the conventional boundary or the trial sequential monitoring boundary

Figure S6: forest plots and TSA on secondary outcomes.

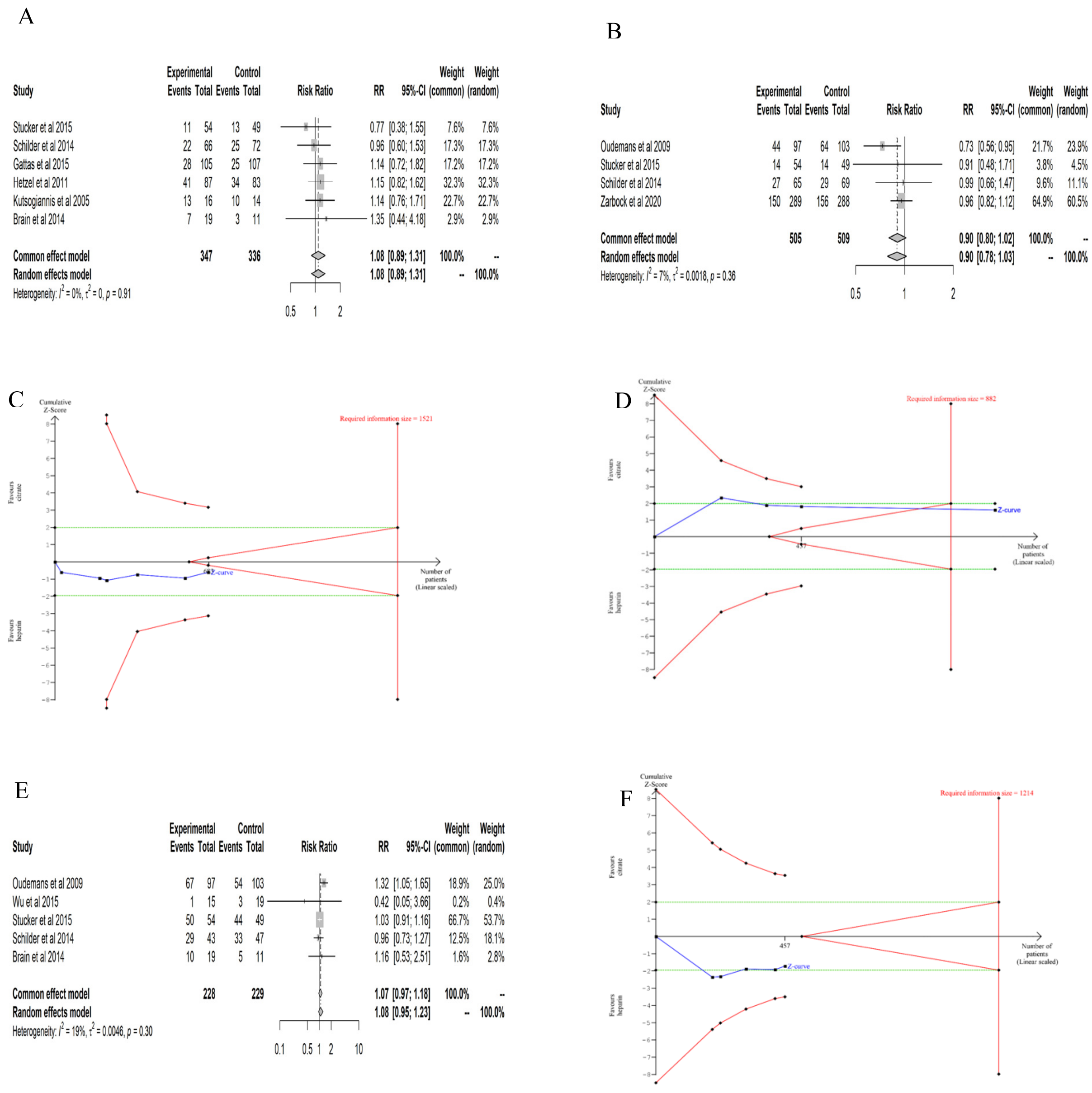

A: fixed-effect model on 28d mortality a RR of 1.08 [0.89,1.31]; p=0.424 was found on 28 days of mortality in the citrate group versus heparin group.

B: fixed-effect model on 90-day mortality a RR of 0.90 [0.80,1.02]; p=0.110 was found on 90-day mortality in citrate group versus heparin group.

C: fixed-effect model of trial sequential analysis for 28d-mortality. A diversity-adjusted information size of 1521 participants calculated based on a 28d-mortality rate of 32.74% in the heparin group, relative risk reduction 20%, alpha=5% (two sided), beta=20%, I^2=0%. The cumulative Z-curve does not cross the futility boundary.

D: fixed-effect model of trial sequential analysis for 3-months mortality. A diversity-adjusted information size of 882 participants calculated on the basis of a 3-months mortality rate of 51.67% in the heparin group, relative risk reduction 20%, alpha=5% (two sided), beta=20%, I^2=17.08%. The cumulative Z-curve crosses the futility boundary and enters the futility area.

E: fixed effects model of renal recovery a RR of 1.07 [0.97,1.18]; p=0.176 between the citrate group and the heparin group.

F: fixed-effect model of trial sequential analysis for renal recovery. A diversity-adjusted information size of 1214 participants calculated based on a renal recovery rate of 60.70% in the heparin group, relative risk reduction 20%, alpha=5% (two sided), beta=20%, I^2=56.54%.
